# Adenosine and ATPγS protect against bacterial pneumonia-induced acute lung injury

**DOI:** 10.1038/s41598-020-75224-0

**Published:** 2020-10-22

**Authors:** Christine M. Gross, Anita Kovacs-Kasa, Mary Louise Meadows, Mary Cherian-Shaw, David J. Fulton, Alexander D. Verin

**Affiliations:** Pulmonary Division, Vascular Biology Center, Medical College of Georgia, Augusta University, 1459 Laney Walker Blvd, CB 3210-A, Augusta, GA 30912 USA

**Keywords:** Phosphorylation, Chronic obstructive pulmonary disease

## Abstract

Lipopolysaccharide (LPS), a component of the outer membrane of gram-negative bacteria, disrupts the alveolar-capillary barrier, triggering pulmonary vascular leak thus inducing acute lung injury (ALI). Extracellular purines, adenosine and ATP, protected against ALI induced by purified LPS. In this study, we investigated whether these purines can impact vascular injury in more clinically-relevant *E.coli* (non-sterile LPS) murine ALI model. Mice were inoculated with live *E. coli* intratracheally (i.t.) with or without adenosine or a non-hydrolyzable ATP analog, adenosine 5′-(γ-thio)-triphosphate (ATPγS) added intravenously (i.v.). After 24 h of *E. coli* treatment, we found that injections of either adenosine or ATPγS 15 min prior or adenosine 3 h after *E.coli* insult significantly attenuated the *E.coli*-mediated increase in inflammatory responses. Furthermore, adenosine prevented weight loss, tachycardia, and compromised lung function in *E. coli*-exposed mice. Accordingly, treatment with adenosine or ATPγS increased oxygen saturation and reduced histopathological signs of lung injury in mice exposed to *E. coli*. Lastly, lung-targeting gene delivery of adenosine or ATPγS downstream effector, myosin phosphatase, significantly attenuated the *E. coli*-induced compromise of lung function. Collectively, our study has demonstrated that adenosine or ATPγS mitigates *E. coli*-induced ALI in mice and may be useful as an adjuvant therapy in future pre-clinical studies.

## Introduction

Acute respiratory distress syndrome (ARDS) and its less severe form, acute lung injury (ALI), are severe inflammatory disorders of the lung which involve massive leukocyte infiltration, increased vascular permeability and hypoxemia^1,2^. Sepsis associated with pneumonia is a major underlying cause of ALI/ARDS^3^. Lung instillation of gram-negative bacterial species, including *Escherichia coli* (*E. coli*) or purified components of its bacterial wall such as lipopolysaccharide (LPS), are common approaches to induce ALI in animal models^4–6^. While much research has been invested into the mechanisms of ALI, there remains no proven pharmacological approaches and the only treatment that has consistently reduced mortality in ALI patients is protective ventilation with low tidal volume^2^. Clearly, significant gaps in our knowledge remain and a greater understanding of the mechanisms by which gram-negative bacteria promote pulmonary vascular barrier dysfunction is essential to develop more effective therapies and improve the outcomes of patients with ALI.

Purines (ATP, ADP, and adenosine) can be released from the cytoplasm into the extracellular space where they serve as important intercellular signaling molecules that act via cell surface receptors^7^. Normally, extracellular ATP and its final purine metabolite, adenosine, are rapidly degraded to concentrations in the sub nanomolar range; although, these concentrations can temporarily rise into the micromolar range under pathological conditions^8,9^, including sepsis^10^. Purinergic cell signaling is mediated by two families of membrane-bound receptors that are designated P1 and P2 which are distinguished by their ability to recognize adenosine and ATP, respectively^7^. There are four subtypes of the adenosine P1 receptor which are designated A1, A2A, A2B and A3 and they all share the ability to link to G proteins^7^. The P2 receptor family is broadly subdivided into P2X and P2Y receptors^7^. There are seven P2X receptor subtypes, P2X1-P2X7 which act as ATP-gated, non-selective cation channels. In contrast, the eight P2Y receptor subtypes, P2Y1, 2, 4, 6, 11–14, are G protein-coupled receptors that recognize, with varying degrees of affinity, either ATP, ADP, UTP, UDP, or UDP-glucose^7^. The lung is enriched with many different types of purinergic receptors that are expressed in multiple cell types and mediate important functions including pulmonary vasodilation^11^, bronchoconstriction^12,13^, immune cell modulation^14^, ciliary beat frequency^15,16^, and pulmonary surfactant release^17^. We have previously reported that an intravenous bolus of either adenosine 5′-(γ-thio)-triphosphate (ATPγS), a non-hydrolyzable ATP analog^18^, or adenosine^19^ protects against pulmonary inflammation and edema in a sterile model of LPS induced ALI in mice and this is supported by other reports that either extracellular ATP or adenosine provides protection against sepsis^20,21^ and ischemia-reperfusion^22,23^. In addition, the P2Y1 and P2Y2 receptors have been shown to mediate improved survival and reduced pulmonary capillary protein leak in mice infected with the gram-negative bacteria *Pseudomonas aeruginosa*^24^. ATP administration has also been shown to improve survival and reduce bacterial counts in murine models of gram-negative and gram-positive sepsis induced by *E. coli* and *Staphylococcus aureus*^25^, respectively. Similarly, extracellular adenosine has been shown to improve survival and reduced lung bacterial counts in mice exposed to gram-positive *Streptococcus pneumoniae*^26^.

Nevertheless, despite the above supportive findings, it is not yet known whether therapeutic intervention with extracellular purines can improve lung function and hemodynamic parameters in a mouse model of gram-negative bacterial pneumonia. Therefore, this study was undertaken to investigate the pre-clinical efficacy of the purines, adenosine and ATPγS, in limiting *E. coli* pneumonia-induced inflammation and lung injury. We demonstrated that after 24 h of i.t. *E. coli* inoculation, i.v. injections of either adenosine or ATPγS 15 min prior or adenosine 3 h after *E.coli* insult significantly attenuated the *E. coli-*mediated increase in protein extravasation and cells infiltration into the bronchoalveolar lavage fluid (BALF). Furthermore, adenosine prevented weight loss, tachycardia, and compromised lung function in *E. coli*-exposed mice. Accordingly, treatment with adenosine or ATPγS increased oxygen saturation and reduced histopathological signs of lung injury in mice exposed to E. coli. Further, lung-targeting delivery^27^ of constitutively active (C/A) MYPT1^28^, a downstream adenosine and ATPγS effector^29,30^, protected lung function from *E.coli*-induced lung injury. Along with our published^18,19^ results on the protective effects of ATPγS and adenosine in sterile ALI model induced by purified LPS, these data suggested promising pre-clinical potential of these purines for the treatment of ALI/ARDS.

## Materials and methods

### Animals

All animal care and experimental procedures were approved by the Institutional Animal Care and Use Committee of Augusta University (Augusta, GA). Adult male C57BL/6NHsd mice (7–8 weeks; Envigo, Indianapolis, IN) were used in all experiments. Mice were maintained at a room temperature of 23 ± 1 °C and exposed to a 12 h alternating light/dark cycle. The animals were fed standard rodent chow (Teklad no. 2918; Envigo) and given tap water ad libitum.

### Escherichia coli pneumonia induced lung injury model

*Escherichia coli* (*E. coli*) (ATCC® 25922™; Manassas, VA), a clinically isolated strain, were inoculated from one colony on Luria Bertani (LB) agar plates into 5 ml LB broth to grow at 37 °C overnight and then, re-inoculated (1:10) into 10 ml LB broth to grow to mid-logarithmic phase for another 2 h. Bacteria were harvested by centrifugation at 5000 g for 10 min, washed twice in 0.9% saline, re-suspended in 10 ml 0.9% saline, and quantified by measuring the optical density at 600 nm (OD_600_) using a spectrophotometer (1 OD_600_ = 1 × 10^8^ *E. coli*/ml). Mice were anesthetized by intraperitoneal (i.p.) injection of ketamine (100 mg/kg) and xylazine-HCl (10 mg/kg), then neck midline incision was performed, and the trachea and right internal jugular vein (IJV) were exposed. Fifteen minutes before instillation, mice received either adenosine, ATPγS (final calculated plasma concentration 100 μM for both; Sigma-Aldrich, St. Louis, MO), or vehicle (0.9% saline) intravenously (i.v.) through the right IJV, then mice were inoculated intratracheally (i.t) with 1 × 10^5^ bacteria in 30 μl of *E. coli* suspension in 0.9% saline or 30 μl of 0.9% sterile saline. In post-treatment experiments, adenosine was added 3 h after *E.coli* insult in the same fashion as described above. Mice were weighed at the start of the experiment and after 24 h. Mice were sacrificed 24 h after infection by intraperitoneal (i.p.) injection of ketamine (500 mg/kg) and xylazine-HCl (50 mg/kg), then bronchoalveolar lavage fluid (BALF) was collected and analyzed as described below. Lungs were flushed with ice-cold EDTA-1 × PBS and then harvested and fixed in 10% formalin for immuno-histochemical evaluation (described below). Alternatively, 24 h after *E.coli* insult, the mice were anesthetized with an i.p. injection of ketamine (100 mg/kg) and xylazine-HCl (10 mg/kg) and lung function studies were performed as described below.

### Isolation of BALF

BALF was obtained by injecting and aspirating 1 ml 1 × PBS via a tracheal cannula, as we have previously described^31^. The cells in the BALF were precipitated at 2500 g for 10 min, then the supernatant was analyzed for protein content using the BCA Protein Assay (Thermo Fisher Scientific, Rockford, IL). The cell pellet was re-suspended in water for 15 s to lyse the red blood cells, and then the salt concentration was adjusted by the addition of 20 × PBS. The total cell count was determined by a hemocytometer^31^.

### Histopathological analysis of the mouse lung

Lung sections (4 μm) from paraffin-embedded blocks were prepared as we have described^31^, then were cut, mounted on treated slides (Superfrost plus; Fisher Scientific, Pittsburgh, PA), deparaffinized in xylene, and passed through a graded series of alcohol solutions to distilled water^31^. Slides were stained with hematoxylin and eosin (H & E), and then dehydrated, cleared, and mounted with Cytoseal 60 (Thermo Fisher Scientific).

### Myeloperoxidase staining

For myeloperoxidase (MPO) staining lung sections (5 μm) were processed and mounted on the slides as described above, then endogenous peroxidases were quenched with 0.3% H_2_O_2_ for 5 min followed by two rinses with distilled water. MPO staining was performed using anti-MPO antibody (1:100 dilution, Abcam, Cambridge, MA) following by incubation with a secondary peroxidase-labeled polymer conjugated to goat anti-rabbit IgG (Envision + , Dako Corporation) as we have previously described in details^31^. Bound antibody was detected with 3,3′-diaminobenzidine (DAB + substrate kit, Dako Corporation). Slides were counterstained with Mayer’s hematoxylin and then dehydrated, cleared, and mounted with Cytoseal 60 (Thermo Fisher Scientific). To assess the extent of histological lung injury in mice a designated scoring system (Lung Injury Score) published by the American Thoracic Society^32^ was utilized as we have previously described in details^19^.

### Assessment of respiratory mechanics

Parameters of lung function such as pressure–volume curves, transcutaneous oxygen saturation and heart rate were evaluated in anesthetized mice (i.p. injection of ketamine (100 mg/kg) and xylazine-HCl (10 mg/kg) twenty-four hours after *E. coli* exposure as we have previously described^31^. After the measurement of respiratory function, the mice were sacrificed by thoracotomy^33^.

### In vivo DNA delivery into mouse lung endothelium with JetPEI

In vivo gene delivery was performed as we have previously described^27,31^. Briefly, constitutively-active (C/A) MYPT1 in pcDNA 3.1 mammalian expression plasmid or control empty plasmid pcDNA 3.1^28^ (40 μg each) were incubated with glucose and jetPEI reagent (Polyplus-transfection Inc, New York, NY), as per manufactures instruction for 15–30 min. Then, the cDNA/jetPEI complexes were injected into 7–8 week old male mice via the tail vein^27^ and 72 h later mice were treated with *E.coli* and changes in respiratory mechanics were assessed as described above. Expression of MYPT1 was assessed in tissue extracts from snap-frozen lung by immunoblotting with anti-MYPT1 antibody using actin as a loading control^27^.

### Statistical analysis

Statistical analysis was performed based on at least three independent experiments using GraphPad Prism version 5.01 for Windows (GraphPad Software, San Diego, CA). The mean ± SEM was calculated in all experiments, and statistical significance was determined by one-way analysis of variance (for ≥ 3 groups). For the analysis of variance, Newman-Kuels post-hoc testing was employed. A value of *P* < 0.05 was considered significant.

### Ethical issues

All methods were carried out in accordance with relevant guidelines and regulations. Named Augusta University institutional committees approved all experimental protocols.

## Results

**Figure 1:**
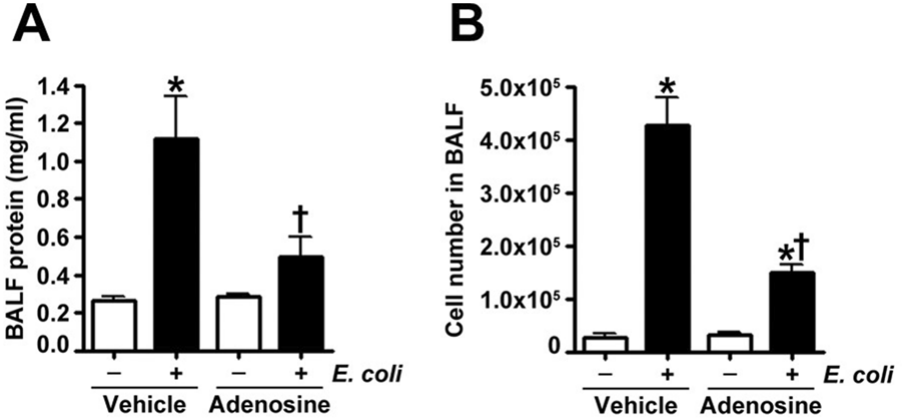
Adenosine pretreatment significantly attenuates *E. coli*-mediated protein extravasation and cell infiltration in BALF in a murine model of ALI. Either adenosine (100 μM) or saline (vehicle) was instilled intravenously 15 min before *E. coli* (intratracheally, for 24 h) challenge. The mice were anesthetized, and the bronchoalveolar lavage fluid (BALF) was collected and centrifuged. (**A**) The protein content (mg/ml) was determined in the resultant supernatants using a BCA protein assay kit. (**B**) The cells were counted using a hemocytometer. Values are mean ± SEM, n = 4–6. *P < 0.05 vs. Vehicle, ^†^*P* < 0.05 vs. Vehicle + *E. coli*.

**Figure 2:**
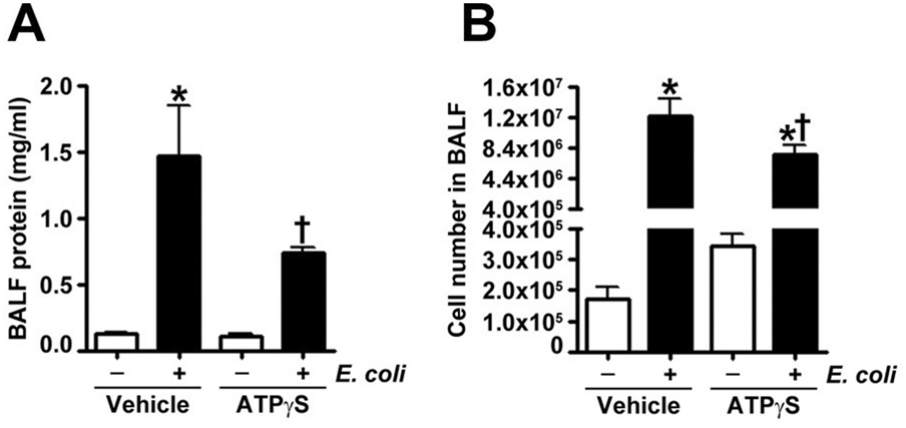
ATPγS pretreatment significantly attenuates *E. coli* mediated protein extravasation and cell infiltration in BALF in a murine model of ALI. Either ATPγS (100 μM), or saline (vehicle) was instilled intravenously 15 min before *E. coli* (intratracheally, for 24 h) challenge. The mice were anesthetized, and the bronchoalveolar lavage fluid (BALF) was collected and centrifuged. (**A**) The protein content (mg/ml) was determined in the resultant supernatants using a BCA protein assay kit. (**B**) The cells were counted using a hemocytometer. Values are mean ± SEM, n = 4–6. *P < 0.05 vs. Vehicle, ^†^*P* < 0.05 vs. Vehicle + *E. coli*.

**Figure 3:**
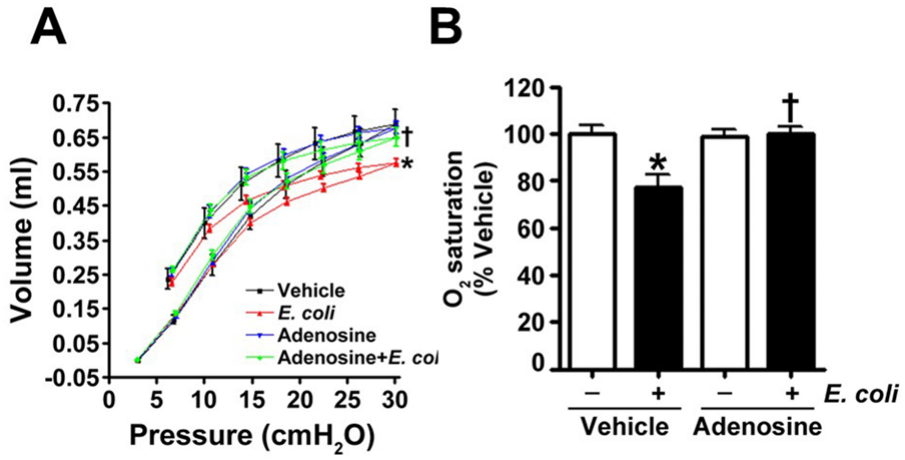
Adenosine attenuates *E.coli*-induced compromise of lung respiratory functions. (**A**) Mice were intravenously injected with either adenosine (100 μM) or saline (vehicle) followed after 15 min by intratracheal inoculation with *E. coli*. Lung mechanics was evaluated in anesthetized mice (i.p. injection of ketamine (100 mg/kg) and xylazine-HCl (10 mg/kg) twenty-four hours after *E. coli* exposure as we have previously described^31^. After the measurement of respiratory function, the mice were sacrificed by thoracotomy^33^. The data represent pressure–volume loops for four groups with two curves: one for inhalation (upper curve) and one for exhalation (lower curve of the same color) events. Values are mean ± SEM, n = 4–6. *P < 0.05 vs. Vehicle, ^†^*P* < 0.05 vs. Vehicle + *E. coli*. (**B**) Mice were intravenously injected with either adenosine (100 μM), or saline (vehicle) followed after 15 min by intratracheal inoculation with *E. coli*. After 24 h, transcutaneous oxygen saturation was monitored via a small animal pulse oximeter by placing the non-invasive sensor on the neck. Values are mean ± SEM, n = 4–6. *P < 0.05 vs. Vehicle, ^†^*P* < 0.05 vs. Vehicle + *E. coli*.

**Figure 4:**
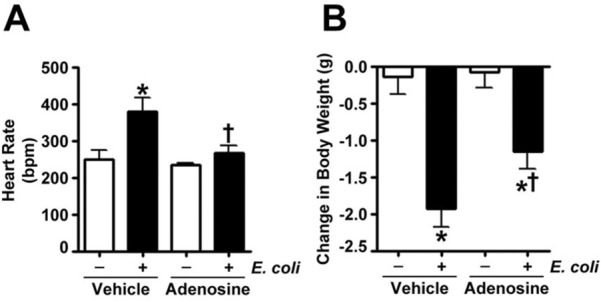
Adenosine prevents the increase in heart rate and weight loss in *E. coli* challenged mice. (**A**) Mice were intravenously injected with either adenosine (100 μM) or saline (vehicle) followed after 15 min by intratracheal inoculation with *E. coli*. After 24 h, heart rate was monitored via a small animal pulse oximeter by placing the non-invasive sensor on the neck. Values are mean ± SEM, n = 4–6. *P < 0.05 vs. Vehicle, ^†^*P* < 0.05 vs. Vehicle + *E. coli*. (**B**) Mice were intravenously injected with either adenosine (100 μM) or saline (vehicle) followed after 15 min by intratracheal inoculation with *E. coli*. Mice were weighed at the start of the experiment and at 24 h before termination of the experiment. Values are mean ± SEM, n = 4–6. *P < 0.05 vs. Vehicle, ^†^*P* < 0.05 vs. Vehicle + *E. coli*.

**Figure 5:**
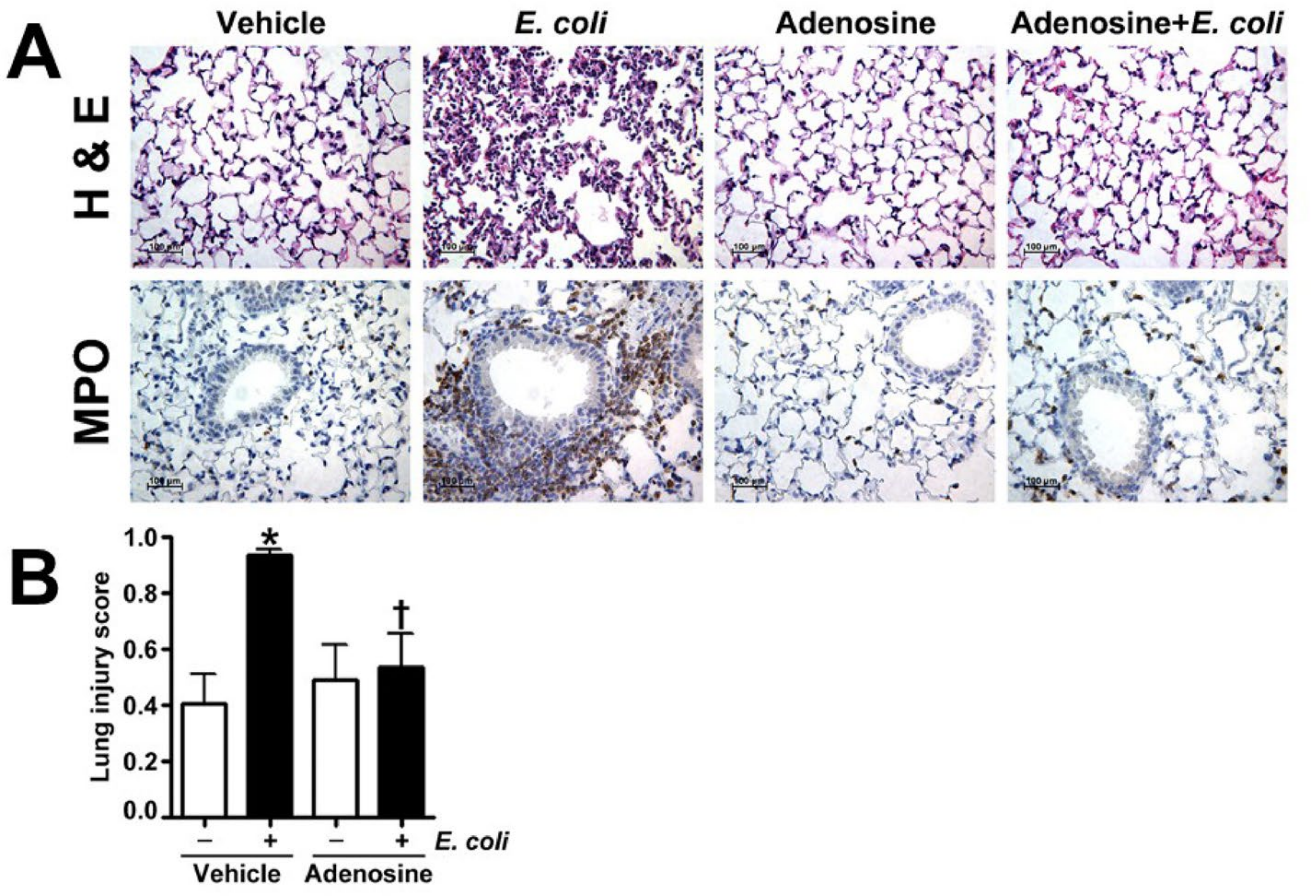
Adenosine pretreatment significantly attenuates lung injury in *E. coli* challenged mice. Mice were intravenously injected with either adenosine (100 μM) or saline (vehicle) followed after 15 min by intratracheal inoculation with *E. coli*. Lung sections were evaluated for inflammatory changes after hematoxylin and eosin staining (**A**, upper panel), neutrophil infiltration after MPO staining (**A**, lower panel), (representative micrographs are shown), and scored for lung injury (**B**). Values are mean ± SEM, n = 4–6. *P < 0.05 vs. Vehicle, ^†^*P* < 0.05 vs. Vehicle + *E. coli*.

**Figure 6:**
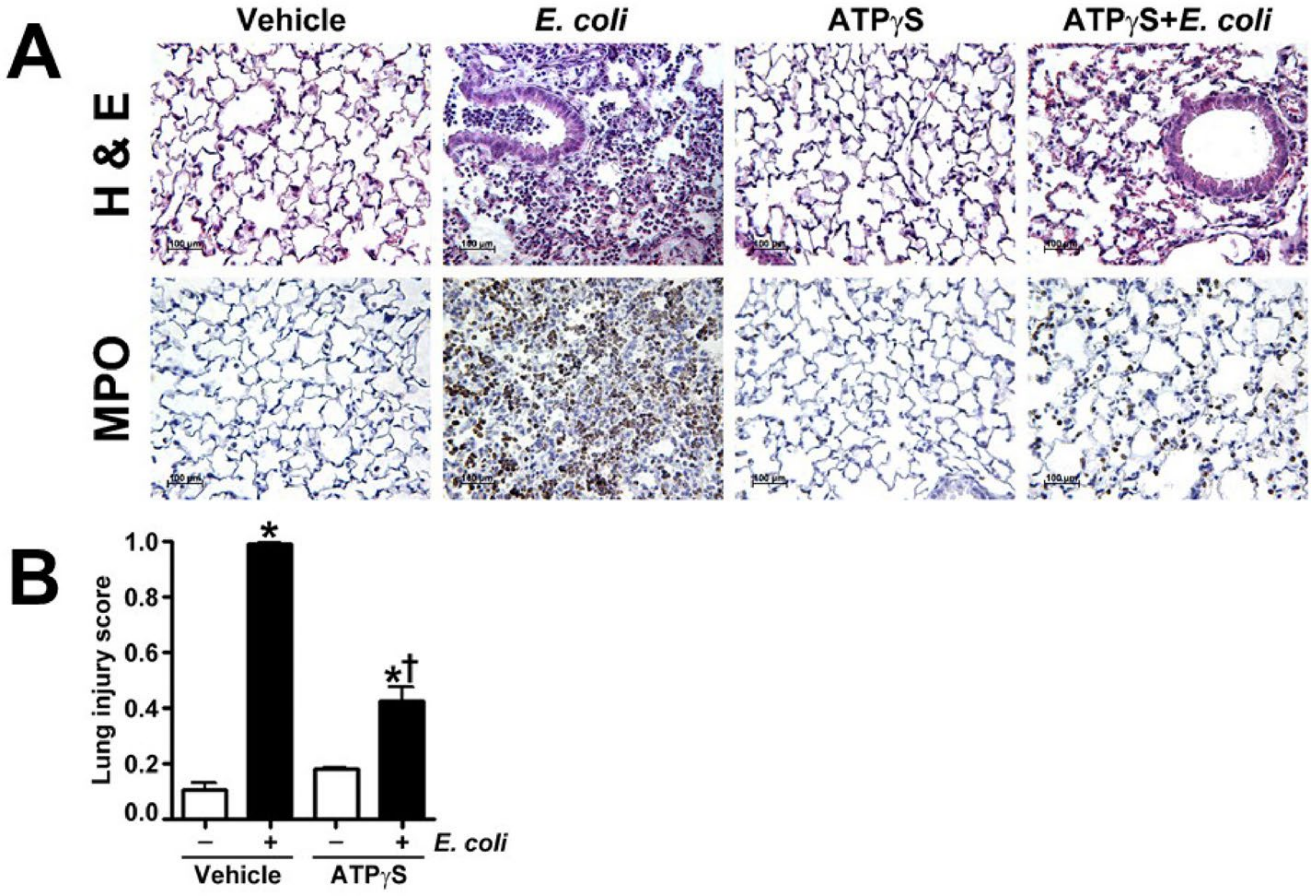
ATPγS pretreatment significantly attenuates lung injury in *E. coli* challenged mice. Mice were intravenously injected with either ATPγS (100 μM) or saline (vehicle) followed after 15 min by intratracheal inoculation with *E. coli*. Lung sections were evaluated for inflammatory changes after hematoxylin and eosin staining (**A**, upper panel), neutrophil infiltration after MPO staining (**A**, lower panel), (representative micrographs are shown), and scored for lung injury (**B**). Values are mean ± SEM, n = 4–6. *P < 0.05 vs. Vehicle, ^†^*P* < 0.05 vs. Vehicle + *E. coli*.

Previous studies have shown that adenosine^19^ and ATPγS^18^ provide protection against a sterile model of LPS-induced lung injury. Therefore, in this study, we further explored whether these purines can limit lung injury associated with bacteria (*E. coli*)-induced pneumonia. Mice were administered intravenous adenosine, ATPγS, or saline (vehicle) which was followed 15 min later by intratracheal inoculation of saline or the *E. coli* (strain ATCC 25922, 1 × 10^5^ bacteria in 30 µl suspension). 24 h after inoculation analysis of the BALF revealed that pretreatment with adenosine (Fig. 1A) or ATPγS (Fig. 2A) reduced *E. coli* stimulated protein extravasation into the airspaces. Consistent with these results, microscopic analysis of the BALF indicated that mice pretreated with adenosine or ATPγS exhibited significantly less cellular infiltration following *E. coli* exposure (Fig. 1B and 2B). Accordingly, adenosine added 3 h after *E.coli* insult significantly attenuated increase in BALF protein and cell count induced by *E.coli* (Supplemental Fig. 1). Analysis of lung mechanics revealed that *E. coli* exposure resulted in a downward displacement of the pressure–volume curve in vehicle treated mice which was not observed in adenosine treated mice in which lung mechanics were preserved (Fig. 3A). Accordingly, adenosine significantly improved oxygen saturation in E. coli inoculated mice (Fig. 3B). In addition, adenosine (Fig. 4A) treatment significantly reduced the tachycardia induced by *E. coli.* Weight loss was observed in mice exposed to *E. coli*, presumably due to anorexia or dehydration. Adenosine (Fig. 4B) pretreatment attenuated the *E. coli* induced weight loss. While the effects of ATPγS on *E.coli*-induced weight loss, lung mechanics and tachycardia have similar response patterns, they were not statistically significant (Supplemental Fig. 2A and 3). In contrary, similar to adenosine (Fig. 2B), ATPγS (Supplemental Fig. 3B) restored oxygen saturation compromised by *E. coli* insult. Histological assessment of lung sections stained with H & E and MPO indicated that treatment with either adenosine (Fig. 5A) or ATPγS (Fig. 6A) attenuated the morphological changes induced by *E. coli* instillation. Indeed, evidence of leukocyte and red blood cell extravasation, hyaline membranes, and proteinaceous debris accumulation in the alveoli were all significantly reduced in the lungs of *E. coli* exposed mice that were treated with either adenosine (Fig. 5 A) or ATPγS (Fig. 6A). In addition, the severity of lung injury was assessed using a semi-quantitative histopathological scoring system^32^ that encompasses the extent of neutrophil infiltration and serum protein accumulation within the airspaces, thickening of the alveolar septa, and the presence of hyaline membranes. The intratracheal instillation of *E. coli* significantly increased the lung injury score in vehicle treated mice but not in either adenosine (Fig. 5B) or ATPγS (Fig. 6B) treated mice.

**Figure 7:**
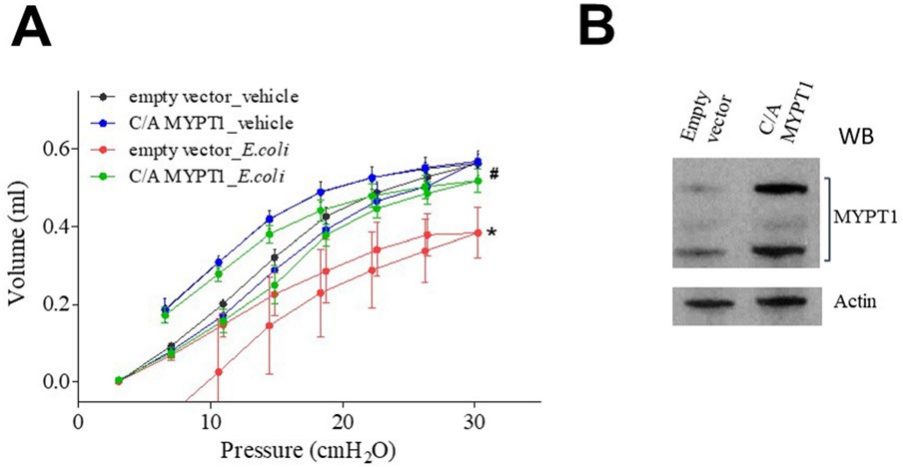
MLCP activity protected lung mechanics in E.coli murine model of ALI. (**A**) Plasmids^28^ encoding C/A MYPT1 or empty pcDNA 3.1 (both 40 μg) were complexed with JetPEI and injected via a tail vein then 72 h later mice were inoculated with live *E.coli* suspension as described above, and pressure/volume curves were analyzed after 24 h of *E. coli* exposure. The data represent pressure–volume loops for four groups with two curves: one for inhalation and one for exhalation events. Values are mean ± SEM, n = 4–6. *P < 0.05 vs. empty vector, ^#^*P* < 0.05 vs. vector + *E.coli*. (**B**) Immunoblotting with anti-MYPT1 antibody^27^ shows the level of MYPT1 overexpression in lung homogenates. Membranes were stained with primary antibody against MYPT1 followed by anti-rabbit secondary antibody^27^. HRP-labeled anti-actin antibody was added simultaneously with secondary antibody.

We have recently shown that both adenosine and ATPγS protect endothelial barrier function via MLCP-mediated mechanisms *in vitro*^29^. In order to evaluate the role of MLCP activity in *E.coli*-induced compromise of lung function we utilized targeting delivery of constitutively active (C/A) MLCP regulatory subunit (MYPT1) into the lung endothelium using jetPEI approach^27,31^. pcDNA-C/A MYPT1 plasmid or empty vector pcDNA plasmid^28^ (both 40 µg), were incubated with glucose and the jetPEI reagent for 15–30 min. Then, the resulting DNA-jetPEI complexes were injected into mice via the tail vein and after 72 h *E.coli* suspension was introduced and lung function was evaluated as we described above. Figure 7 demonstrated that introduction of (C/A) MYPT1 significantly attenuated *E.coli*-induced loss of lung function suggesting the involvement of MLCP activity in lung function preservation in *E.coli*-induced ALI model in mice.

## Discussion

A defining characteristic of pathophysiology of ALI is the massive pulmonary inflammation that compromises the microvascular endothelial barrier leading to the extravasation of protein-rich fluid in alveolar and extra-alveolar spaces^1^. In the current study we have used a clinically relevant, non-sterile mouse model of gram-negative bacterial pneumonia to demonstrate that the intravenous administration of purinergic agonists, either adenosine or ATPγS, provides protection against *E. coli* induced neutrophil infiltration and protein exudation into the lungs, improves oxygen saturation and mitigates lung injury. Adenosine treatment significantly blunted *E. coli* mediated changes in body weight, lung mechanics, and heart rate. This data is in agreement with our previous studies showing that an intravenous bolus of either ATPγS^18^ or adenosine^19^ protects against inflammation and pulmonary edema in a sterile murine model of LPS induced ALI. Although this study does not address the receptor-mediated mechanism underlying adenosine and ATP mediated lung protection, earlier studies by our group have indicated that the A2 receptors are involved in adenosine-induced endothelial barrier enhancement in human pulmonary macro- and microvascular endothelial cells^29,34^. In contrary, ATP and ATPγS were shown to improve human pulmonary endothelial barrier function through the activation of P2Y receptors^29,30^. While disruption of alveolar barrier leading to pulmonary edema is prominent feature of ALI^2^, other factors such as endothelial and epithelial cells apoptosis may contribute to ALI development^35^. Inhibitors of apoptosis improves survival in rodents ALI models^36^. Edemagenic factors, like LPS, produce apoptotic responses in human alveolar epithelial cells^37^. However, whether extracellular purines can oppose *E.coli*-induced ALI via inhibition of apoptosis is unknown and may be subject for future studies.

The adenosine A2 receptor is a G-protein coupled receptor linked to Gαs which stimulates adenylyl cyclase, resulting in cAMP accumulation following by protein kinase A (PKA) activation^34^. In human lung macrovascular and microvascular EC (HPAEC and HLMVEC, respectively), the activation of cAMP-dependent signaling pathways results in improved barrier function^29,34^. In contrary, ATPγS-induced EC barrier enhancement in both cell types involves Gi-mediated cAMP-independent PKA activation^29^. The mechanisms of cAMP-independent PKA activation are ill defined, but our recently published data suggested that they may involve interaction of PKA with PKA-anchoring protein 2 (AKAP2)^29^. Both, adenosine- and ATPγS-induced EC barrier enhancement involve activation of MLCP leading to inhibition of EC contractile responses^29,34^. Further, MLCP is directly involved in lung vascular barrier protection against LPS-induced ALI in murine model^27^. Consistent with these data, our results demonstrated the involvement of MLCP activity in the protection of lung function against *E.coli*-induced lung injury highlighting the key role of MLCP activation in the lung function preservation in both sterile and non-sterile ALI models.

Several studies have shown that extracellular ATP is protective in ALI. In *Pseudomonas aeruginosa* infected P2Y1/P2Y2^−/−^ mice, investigators found diminished survival and elevated protein content in the alveolar spaces, suggesting that P2Y1 and P2Y2 are important for maintaining the alveolar-capillary barrier^24^. An intravenous infusion of ATP improved systemic and pulmonary hemodynamics, cardiac output, stroke volume, arterial blood gases, pH, lung mechanics, and survival in mechanically ventilated piglets with sepsis caused by the gram-positive Group B β-hemolytic streptococci^38^. In a recent study, an intraperitoneal injection of ATP protected mice from *E. coli* and *Staphylococcus aureus* mediated mortality and reduced bacterial counts in peritoneal lavage^25^. Improved survival was found to be dependent on P2X7 receptors which signal to repress the inflammasome-dependent activation of Caspase-1, and was mediated by intact ATP and not its degradation products^25^. Similarly, ATP administration was found to be protective in a mouse model of polymicrobial sepsis with the authors reporting that P2X7 receptor signaling on macrophages was crucial for mediating bacterial killing and reducing pulmonary inflammation^20^. In contrast to these studies, others have shown that P2X7^−/−^ mice exposed to LPS exhibited improved lung mechanics, reduced pulmonary neutrophil infiltration, and improved histopathological findings including less alveolar collapse and fiber deposition^39^. These studies highlight potential differences between sterile and non-sterile models of ALI and stress the importance of performing studies in multiple animal models. In addition, these results imply that ATP can have both physiological and pathological roles during the resolution of an infection. Purines, including ATP and adenosine, are generally considered “danger molecules” that are released to the extracellular space at sites of tissue injury to activate circulating immune cells and stimulate migration towards the site of injury. Accordingly, these purinergic nucleotides play an important role in resolving an active infection but can also cause excessive or chronic inflammation^14^. Along these lines, extracellular ATP has been shown to induce chemokine secretion and neutrophil chemotaxis^25^, which is consistent with the findings of others and is likely P2Y receptor mediated^40,41^. In addition, ATP may be involved in the formation of inflammasomes by binding to P2X7 receptors and promoting inflammatory responses^42,43^.

These findings contrast those of our present study and past publications^18^, which collectively found that intravenous ATPγS administration reduced total leukocyte and neutrophil infiltration in the *E. coli* and LPS challenged mouse lung, which suggests that ATP restrains leukocyte migration. The reasons for these differences are unclear, but may be attributed to: (1) the method of ATP/ATPγS administration: an intraperitoneal injection of ATP will first enter the lymphatic circulation and travel through the lymph nodes eliciting an immune response before ultimately entering into the bloodstream, while an intravenous delivery route, as used in our study, will first pass the pulmonary endothelium where ATP may strengthen barrier function; and (2) the ATPγS we employed is essentially non-hydrolyzable. The latter point is important as numerous studies suggest that inflammasome assembly is dependent upon ATP hydrolysis and ATPase activity^44–46^ and thus it may be assumed that any pro-inflammatory effects of ATP will be much lower with the non-hydrolyzing ATPγS. In fact, we have previously shown that intratracheal introduction of ATPγS itself did not produce any significant inflammatory responses and reduced inflammation and permeability induced by LPS in murine ALI model^18^. ATPγS would also be expected to activate a reduced spectrum of purinergic receptors compared to ATP, which will be hydrolyzed to both ADP and adenosine, which act on distinct types of purinergic receptors. Additional data in support of our findings are that ATP can inhibit the release of pro-inflammatory cytokines IL-12, TNF-α, and IFN-γ and increase the production of IL-10, an anti-inflammatory cytokine, in LPS exposed macrophages^47^.

While controversy remains over the cell type and receptor(s) involved, adenosine administration has been shown to be protective in many models of ALI^19,21,23,48–55^. Indeed, our present conclusion that intravenous adenosine attenuates *E. coli* induced lung injury are most directly supported by a study showing that intratracheal instillation of the selective A2A receptor agonist, GW328267C in *E. coli* exposed rats, results in attenuated pulmonary vascular fluid and protein leak, reduced neutrophil infiltration, and improved alveolar fluid clearance^50^. Although, we found that adenosine lowered heart rate and improved oxygen saturation in *E. coli* infected mice, the authors reported a decrease in MAP and arterial pO_2_ with no change in heart rate 3 h after *E. coli* exposure, none of which were altered by GW328267C^50^. The reasons for these differences are unclear but may be related to the different time-points used in the experiments. Multiple receptors may also play a role and intravenous 2-chloroadenosine, an A1 receptor agonist, reduces *E. coli* induced protein leak, leukocyte infiltration, and TNF-α plasma levels in neutropenic guinea pigs^56^. Similarly, extracellular adenosine has been shown to improve survival, reduce lung bacterial loads and septicemia, and attenuate neutrophil infiltration in the lungs of mice exposed to *Streptococcus pneumoniae*^26^. Interestingly, in this study the authors showed that extracellular adenosine prevented neutrophil transmigration specifically across the pulmonary endothelial barrier, but not through the alveolar epithelium, during infection with *Streptococcus pneumoniae*^26^. It is unclear whether intravenous adenosine administration attenuates neutrophil migration primarily via adenosine receptors on the endothelium or on the neutrophils, and further studies are required to identify the specific receptor(s) involved in neutrophil transmigration. As adenosine has been shown to regulate neutrophil migration directly^57^ as well as reduce endothelial cytokine release and expression of adhesion molecules^58^ both mechanisms are likely involved. Indeed, some studies suggest that extracellular adenosine may impair pathogen clearance during infection. The absence of the A2B receptor in mice improves survival and reduces bacterial loads during *Klebsiella pneumoniae* infection^59^ and polymicrobial sepsis^60^. This data is opposed by studies that have investigated polymicrobial sepsis and found that mice lacking the A2B receptor mice had reduced survival with no change in bacterial clearance^21^ and that CD73-/- mice, which are deficient in an enzyme important for the generation of extracellular adenosine, which also had reduced survival but increased bacterial loads^61^. Together these contrasting findings point to a complex role of adenosine during bacterial infection and indicates the need for additional studies.

In conclusion, our data demonstrates that the purinergic agonists, adenosine and ATPγS, given intravenously can attenuate lung injury induced by *E. coli* pneumonia and this occurs via a reduction in inflammation and pulmonary microvascular protein leak. Based on these findings, we speculate that purinergic receptor modulation may pave the way for future pre-clinical studies on the protective role of extracellular purines in ALI. Further studies will be required to elucidate the mechanisms of adenosine and ATPγS mediated lung protection and to clarify the role of individual purinergic receptors during bacterial pneumonia.

## Supplementary information

Supplementary Information
