# Supplementary material for: Adenosine and ATPγS protect against bacterial pneumonia-induced acute lung injury
Source: Sci Rep. 2020 Oct 22;10:18078. doi: 10.1038/s41598-020-75224-0 (PMC7581771; doi:10.1038/s41598-020-75224-0)

Adenosine and ATPγS Protect Against Bacterial Pneumonia-induced Acute Lung Injury

Christine M. Gross*

Anita Kovacs-Kasa*

Mary Louise Meadows

Mary Cherian-Shaw

David J. Fulton

Alexander D. Verin

From the Vascular Biology Center and Pulmonary Division, Medical College of Georgia,

Augusta University, Augusta, GA, United States of America

Running Head: Adenosine and ATPγS protect against *E. coli* induced ALI

Please address correspondence and proofs to:

Alexander D. Verin, Ph.D.

Vascular Biology Center

Augusta University

1459 Laney Walker Blvd

CB 3210-A

Augusta, GA 30912

Phone: 706-721-1531

Fax: 706-721-9799

Email: averin@augusta.edu

* Authors with an equal contribution

Supplemental Figure 1. Mice were injected intratraheally with *E.coli* 1x10^5 bacteria in 30 µl of *E. coli* suspension in 0.9% saline or 30 µl of 0.9% sterile saline. 3 hours after *E.coli* administration adenosine (final calculated plasma concentration 100 µM) or saline was injected into the right jugular vein. 18 hours later mice were sacrificed and BALF was collected. **A:** The protein content (mg/ml) was determined in the resultant supernatants using a BCA protein assay kit. Values are mean ± SEM, n=5. *P<0.05 vs. Vehicle, #P<0.05 vs. Vehicle+*E. coli*. **B:** The cells were counted using a hemocytometer. Values are mean ± SEM, n=5. ***P<0.05 vs. Vehicle, *P<0.05 vs. Vehicle+*E. coli*.

Supplemental Figure 2. Effect of ATPγS on *E.coli*-induced compromise of lung respiratory functions.

Panel A. Mice were intravenously injected with either ATPγS (100 µM) or saline (vehicle) followed after 15 min by intratracheal inoculation with *E. coli*. Lung mechanics was evaluated in anesthetized mice (i.p. injection of ketamine (100 mg/kg) and xylazine-HCI (10 mg/kg) twenty-four hours after *E. coli* exposure as we have previously described[31]. After the measurement of respiratory function, the mice were sacrificed by thoracotomy[33]. The data represent pressure-volume loops for four groups with two curves: one for inhalation (upper curve) and one for exhalation (lower curve of the same color) events. Values are mean ± SEM, n = 4-6. **Panel B.** Mice were intravenously injected with either adenosine (100 µM), or saline (vehicle) followed after 15 min by intratracheal inoculation with *E. coli*. After 24 h, transcutaneous oxygen saturation was monitored via a small animal pulse oximeter by placing the non-invasive sensor on the neck. Values are mean ± SEM, n = 4-6. *P<0.05 vs. Vehicle, †P<0.05 vs. Vehicle+*E. coli*.

Supplemental Figure 3. Effect of ATPγS on the increase in heart rate and weight loss induced by *E. coli*.

Panel A. Mice were intravenously injected with either ATPγS (100 μM) or saline (vehicle) followed after 15 min by intratracheal inoculation with *E. coli*. After 24 h, heart rate was monitored via a small animal pulse oximeter by placing the non-invasive sensor on the neck. Values are mean ± SEM, n = 4-6. **Panel B.** Mice were intravenously injected with either adenosine (100 μM) or saline (vehicle) followed after 15 min by intratracheal inoculation with *E. coli*. Mice were weighed at the start of the experiment and at 24 h before termination of the experiment. Values are mean ± SEM, n = 4-6. *P<0.05 vs. Vehicle

Supplemental Figure 4. Full image of immunoblotting presented on Figure 7B.

Supplemental Figure 1

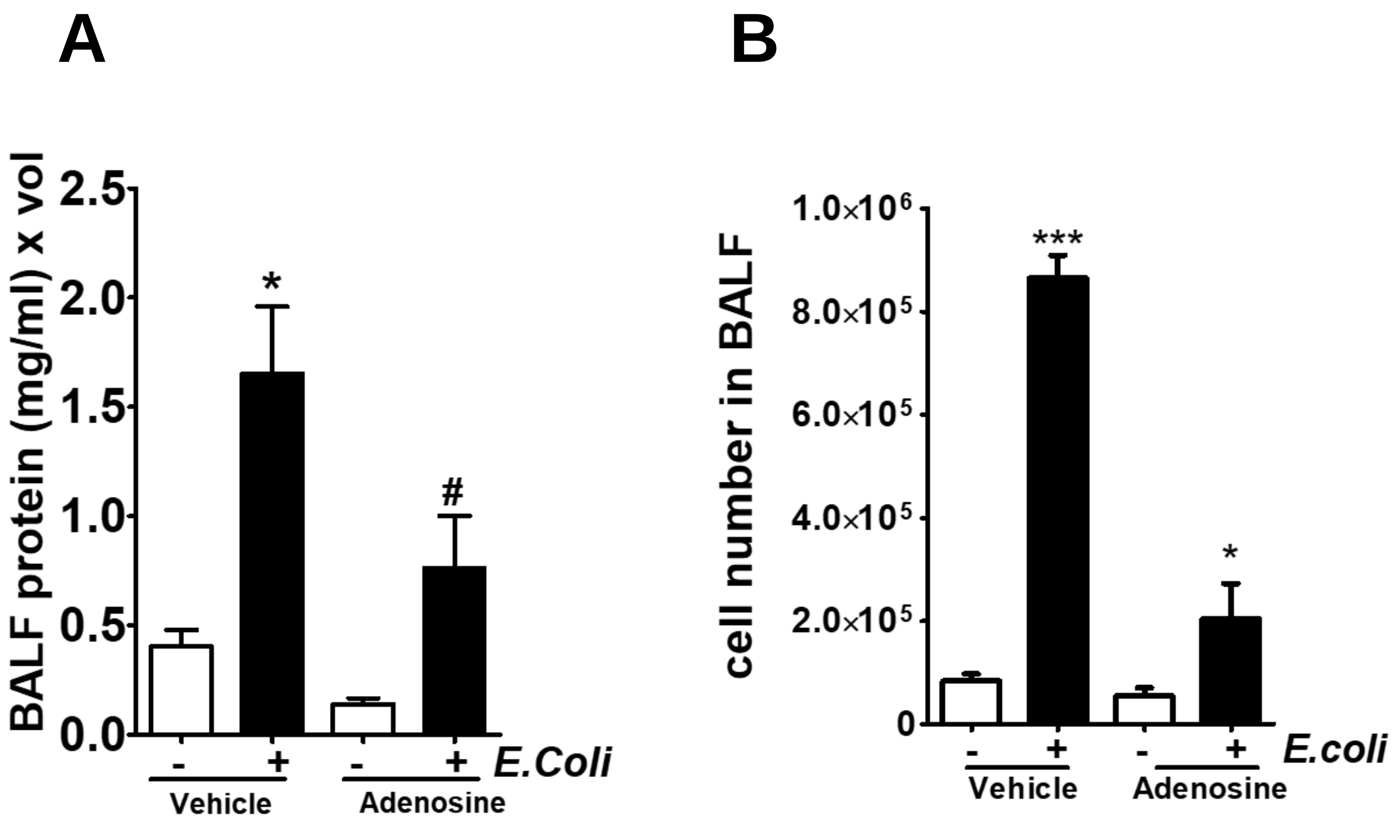

Supplemental Figure 2

A

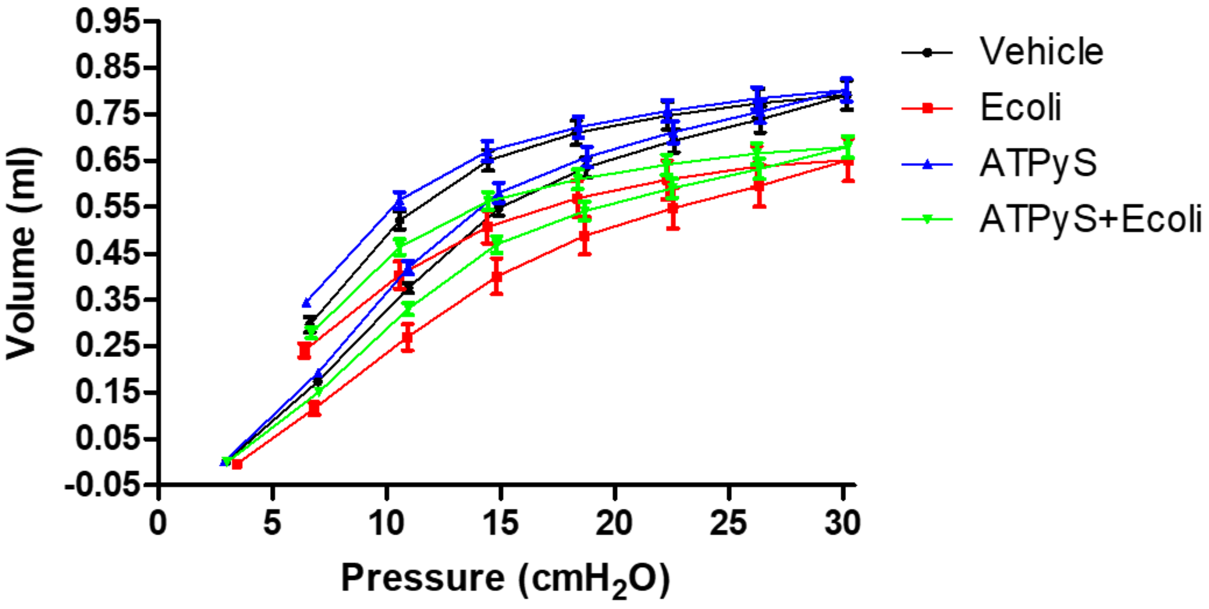

B

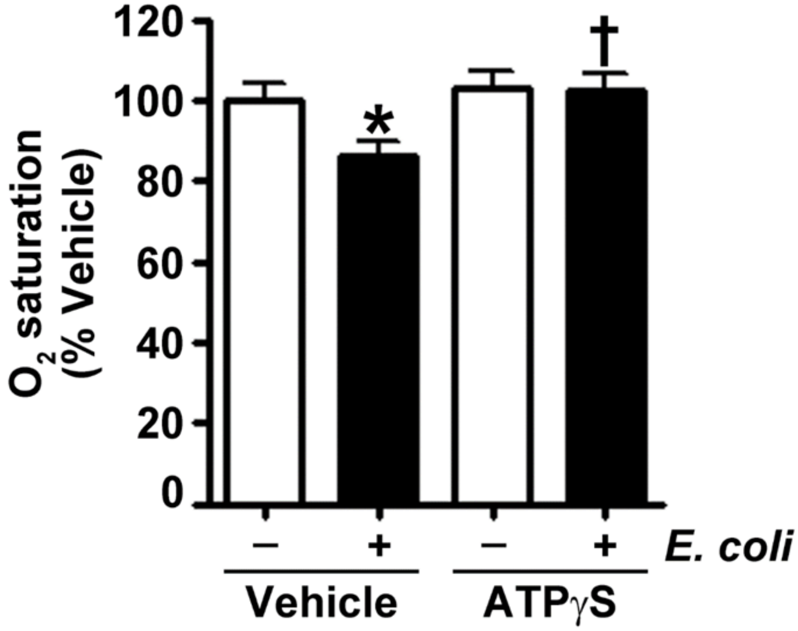

Supplemental Figure 3

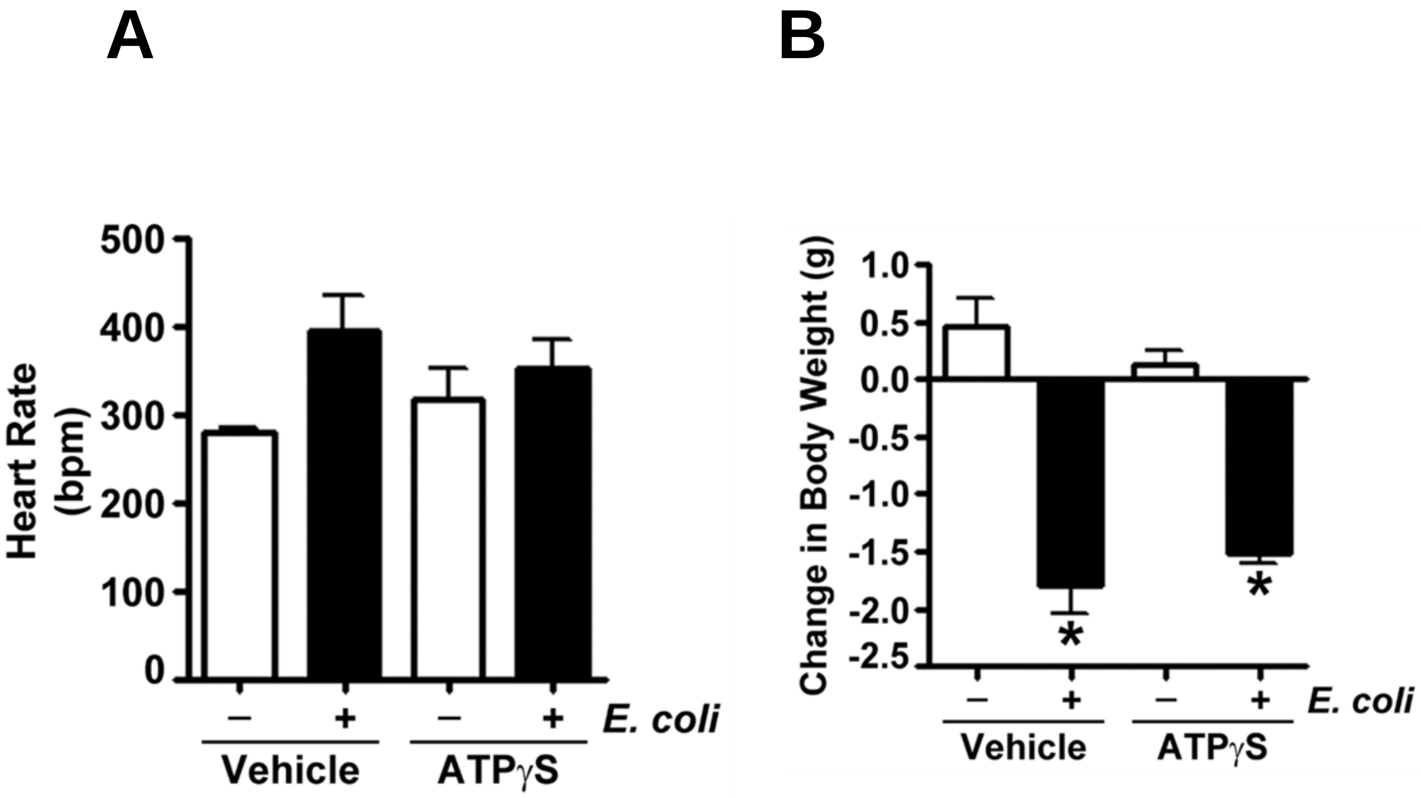

Supplemental Figure 4

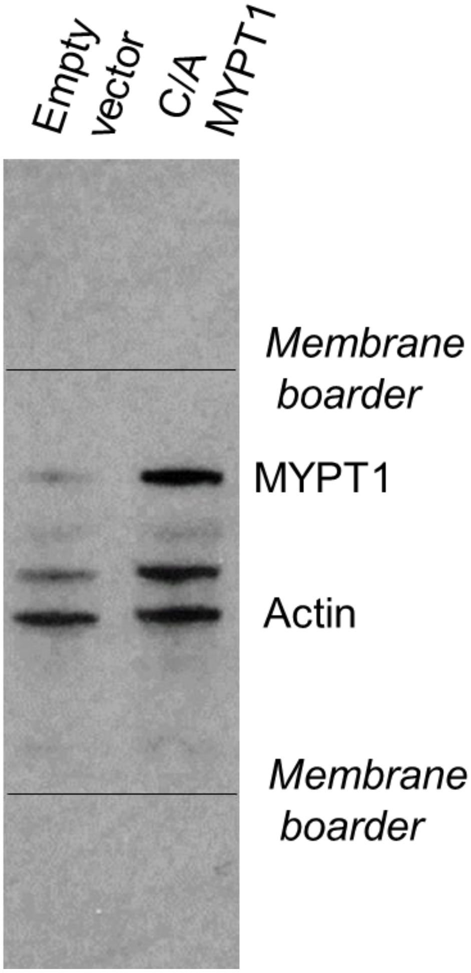

Supplement: Supplementary file 1 — Supplementary Information [file 41598_2020_75224_MOESM1_ESM.pdf]
